# Dominance and G×E interaction effects improve genomic prediction and genetic gain in intermediate wheatgrass (*Thinopyrum intermedium*)

**DOI:** 10.1002/tpg2.20012

**Published:** 2020-03-19

**Authors:** Prabin Bajgain, Xiaofei Zhang, James A. Anderson

**Affiliations:** ^1^ Department of Agronomy & Plant Genetics University of Minnesota St. Paul MN USA; ^2^ The Alliance of Bioversity International and International Center for Tropical Agriculture Cali Colombia

## Abstract

Genomic selection (GS) based recurrent selection methods were developed to accelerate the domestication of intermediate wheatgrass [IWG, *Thinopyrum intermedium* (Host) Barkworth & D.R. Dewey]. A subset of the breeding population phenotyped at multiple environments is used to train GS models and then predict trait values of the breeding population. In this study, we implemented several GS models that investigated the use of additive and dominance effects and G×E interaction effects to understand how they affected trait predictions in intermediate wheatgrass. We evaluated 451 genotypes from the University of Minnesota IWG breeding program for nine agronomic and domestication traits at two Minnesota locations during 2017–2018. Genet‐mean based heritabilities for these traits ranged from 0.34 to 0.77. Using four‐fold cross validation, we observed the highest predictive abilities (correlation of 0.67) in models that considered G×E effects. When G×E effects were fitted in GS models, trait predictions improved by 18%, 15%, 20%, and 23% for yield, spike weight, spike length, and free threshing, respectively. Genomic selection models with dominance effects showed only modest increases of up to 3% and were trait‐dependent. Cross‐environment predictions were better for high heritability traits such as spike length, shatter resistance, free threshing, grain weight, and seed length than traits with low heritability and large environmental variance such as spike weight, grain yield, and seed width. Our results confirm that GS can accelerate IWG domestication by increasing genetic gain per breeding cycle and assist in selection of genotypes with promise of better performance in diverse environments.

**Abbreviations**
BL: Bayesian LASSO
BRR: Bayesian ridge regression
GBLUP: Genomic best linear unbiased prediction
GEBV: Genomic‐estimated breeding value
GS: Genomic selection
G×E: Genotype by environment interaction
IWG: Intermediate wheatgrass
LD: Linkage disequilibrium
QTL: Quantitative trait locus
SNP: Single nucleotide polymorphism
TKW: Thousand kernel weight

## 1 INTRODUCTION

Predicting the genetic potential of an individual in a breeding population using genome‐wide selection is a powerful method to increase selection efficiency in plant and animal breeding programs (Hayes & Goddard, 2010; Heffner, Lorenz, Jannink, & Sorrells, 2010). This method, commonly known as genomic selection (GS), evaluates a population under selection by estimating its genomic‐estimated breeding values (GEBVs) using whole genome information and statistical functions commonly known as models. For complex quantitative traits controlled by a large number of genes with medium to small effect, GS can be quite effective relative to marker assisted selection alone (Jannink, Lorenz, & Iwata, 2010; Lorenz et al., 2011). In recent years, GS has been a popular tool in crop improvement programs including perennial species (Cros et al., 2015; Fè et al., 2015; Biazzi et al., 2017) to improve a wide range of traits such as yield and domestication traits (Resende et al., 2012; Annicchiarico et al., 2015; Zhang et al., 2016).

Crop domestication and enhancement has been practiced by humans for several thousand years. All current crops and livestock are direct results of active domestication practices. Nearly all of our current crops are of annual growth habit as they occupy 70% of the landscape (FAO, 2013). While annual crops are highly productive and provide a large portion of human nutrition, current agricultural management practices of annual crops also tend to weaken soil and water health (Bestelmeyer et al., 2015). Perennial cropping systems have been proposed as restorative means to nurture soil, water, and air health, as well as for their dual use potential: food and sustainability (Cox, Van Tassel, Glover, DeHaan, & Cox, 2006; de Oliveira, Brunsell, Sutherlin, Crews, & DeHaan, 2018). Compared to their annual counterparts, perennial crops are reported to maintain topsoil 50‐fold more effectively, lower nitrogen losses by 30‐ to 50‐fold, and sequester significantly more carbon per unit area (Gomiero, 2016; Jungers, DeHaan, Mulla, Sheaffer, & Wyse, 2019). As the world population is projected to reach 9.6 billion by 2050 and annual cereal grain production needing to be increased by > 40% (Tripathi, Mishra, Maurya, Singh, & Wilson, 2019), alternative means to produce more food while preserving renewable resources is a pressing need. Intermediate wheatgrass (IWG) can be one such alternative crop that nurtures environmental sustainability while providing food. A perennial cool grass species of the same tribe Triticeae as wheat, IWG domestication was initiated in the 1980s (Wagoner, 1990) with active breeding efforts ongoing at The Land Institute (Salina, KS, USA), Lund University (Lund, Sweden), University of Manitoba (Winnipeg, Canada), University of Minnesota (St. Paul, MN, USA), and USDA‐ARS (Logan, UT, USA).

The University of Minnesota IWG breeding program aims to develop germplasm with higher grain yield, larger seed size, reduced shattering, and improved free grain threshing (percent de‐hulled seeds). Initiated in 2011, the breeding program uses two‐year recurrent selection cycles for trait improvement with substantial reliance on GS. In our current GS approach, 8–10 random half‐sibs from approximately 70 families are evaluated at two locations for multiple agronomic traits. Phenotypic data are used to train GS models that are applied to a population of several thousand seedlings from which the best 70–100 are selected as parents for the next breeding cycle. Using this approach, we have been able to significantly improve grain yield, seed‐related traits, disease resistance, and domestication traits such as shatter resistance and free grain threshing (Zhang et al., 2016; Bajgain et al., 2019a; Bajgain et al., 2019b). Implementation of GS in our program has had a direct impact in notably improving genetic gain in IWG because of better population performances observed in each of our breeding cycles.

**Core Ideas**

- Intermediate wheatgrass is a cross‐pollinated perennial crop with nutritious grain and excellent ecosystem services.
- Genomic selection based breeding has led to germplasm improvement and variety development.
- Using dominance effects in genomic prediction models led to modest increases in trait predictions.
- Use of G×E effects in genomic prediction models significantly increased their ability to predict nearly all traits.
- Cross‐environment predictions varied by trait and location yet can help select high‐heritability traits.

A vast majority of the existing GS models and application tools focus on exploiting additive effects only. The earliest GS models were implemented in dairy cattle breeding programs that sought to select highly performing sires by estimating the additive genetic effects of genome‐wide markers (Meuwissen, Hayes, & Goddard, 2001; VanRaden, 2008). While efficient and predictive, additive‐only models tend to ignore a significant amount of leftover underlying genetic architecture, particularly in the aspect of complex traits, and can cause the problem of ‘missing heritability’ (Eichler et al., 2010). While the extent of control that dominance and higher order genetic interactions have on IWG traits is unknown, some level of heterosis could be expected to affect trait performance in open‐pollinating species, especially in the early generations of synthetic populations (Falk, Rakow, & Downey, 1998; Pembleton et al., 2015). Therefore, using all available genetic information in genomic prediction can improve predictive ability and help geneticists and breeders improve the germplasm.

The IWG breeding populations at the University of Minnesota are evaluated at two Minnesota locations—Crookston and St. Paul—that differ in the amount of precipitation received, day length, temperature, and soil type. Naturally, large genotype by environment (G×E) effects are observed in the trait data each year. Typically, predictions are carried out with best linear unbiased estimates (BLUEs) estimated across all locations and years and/or by using models that do not consider G×E effects. Instead of ignoring the G×E interaction effect, using it to train genomic selection models can boost trait predictability. Burgueño, de los Campos, Weigel, and Crossa (2012) was among the first to demonstrate that higher prediction accuracy can be gained by applying a genomic best linear unbiased prediction (GBLUP) model to multi‐environment data. Subsequently, incorporation of G×E effects, especially multi‐location, multi‐year, and multi‐treatment data have shown substantial improvements in improving prediction accuracies in crops (Crossa et al., 2013; Heslot, Akdemir, Sorrells, & Jannink, 2014; Lopez‐Cruz et al., 2015).

The primary objective of this study was to evaluate multiple GS models for their effectiveness in IWG trait predictions and recommend the overall best model. We mainly tested two model types: Bayesian and GBLUP, and derived their variants with either additive effects only or with both additive and dominance effects. Further, we explored how trait predictions changed when genotype by environment interaction (G×E) effects were implemented in these models and examined cross‐environment trait predictions. We also report the genetic variance components and heritability estimates for all traits, estimated genetic gain based on genomic predictions, and provide a brief outlook on GS‐based IWG breeding.

## 2 MATERIALS AND METHODS

### 2.1 Plant population, phenotyping, genotyping

The intermediate wheatgrass (IWG) plant population used in this study has previously been described in detail (Bajgain et al., 2019a). Briefly, 451 genets from the third IWG recurrent breeding cycle at the University of Minnesota (UMN_C3 hereafter) were used. The term ‘genet’ identifies a genetically unique organism in an outcrossing plant species such as IWG (Zhang et al., 2016). These genets were genotyped using genotyping by sequencing (Poland, Brown, Sorrells, & Jannink, 2012) resulting in 8,899 genome‐wide SNP markers. The population was phenotyped for multiple agronomic traits in St. Paul, MN in 2017 (StP17) and 2018 (StP18), and in Crookston, MN in 2018 (Crk18). In this study, we will focus on spike weight, spike length, number of spikelets per spike, yield, seed mass measured in terms of thousand kernel weight (TKW), seed length, seed width, shatter resistance, and free threshing (i.e. % de‐hulled seeds or ‘threshability’ hereafter) in this study. Data collection on all traits except shatter resistance and threshability have also been previously described (Bajgain et al., 2019a).

Shatter resistance was measured by assessing florets and spikelets breakage from mature spikes. Measurements were taken from three spikes per genet on a 0–4 scale where 0.01 was assigned for genets with no shattering, 0.5 for genets with shattering of up to 10%, 1 for 10–20%, 2 for 20–50%, 3 for 50–80%, and 4 for 80–100% shattering. Threshability was measured as the proportion of naked or de‐hulled seeds after mechanical threshing. This was also measured on a 0–4 scale where 0.01 was assigned for genets with 100% hulled seeds (i.e. no naked seeds), 0.5 for genets with up to 10% de‐hulled seeds, 1 for 10–20%, 2 for 20–50%, 3 for 50–80%, and 4 for 80–100%. Phenotypic data were adjusted for trial effects using the method outlined by Sallam, Endelman, Jannink, and Smith (2015) to obtain BLUEs (PHENO_adj_) for statistical analyses. Briefly, trial effects were first estimated for all traits by treating trial as fixed effects in a mixed model equation in the MIXED procedure in SAS 9.4. BLUEs for each genet were calculated in the next step by correcting for the trial effect estimated in the previous step.

### 2.2 Variance components and heritability estimates

Variance components for each trait were estimated using the average information (AI) restricted maximum likelihood (REML) algorithm (Gilmour, Thompson, & Cullis, 1995) that uses a univariate mixed model of the following form:

$$Y=X_{\beta }+Z_{\mu }+\varepsilon$$

where **Y** is the vector of adjusted phenotypic data (i.e. PHENO_adj_); **X** and **Z** are incidence matrices for fixed and random effects, respectively; β is a vector of fixed effects; μ is a vector of random effects; and ε is the residual variance.

Variance components were estimated with two major model types using: only additive effects (A) and with both additive and dominance effects (AD). The model above was therefore extended to following variants:

$Y_{A}=X_{\beta }+Z_{\mu }a+\varepsilon$ when only additive effects were included in the model (A)
$Y_{\mathrm{AD}}=X_{\beta }+Z_{\mu }a+Z_{\mu }d+\varepsilon$ when additive and dominance effects were included in the model (AD)

In an attempt to include higher order genetic effects such as epistasis and interactions among additive, dominance, and epistasis effects, an additional extension of these models that would consider all additive, dominance, and residual genetic effects (ADE) was briefly considered. However, the additive, dominance, and epistatic effects in such ADE model are not orthogonal, and therefore the partition of genetic effects becomes more challenging (Covarrubias‐Pazaran, 2016; Vitezica, Legarra, Toro, & Varona, 2017). Therefore, only the A and AD matrices were implemented in all genomic prediction models. Model fitness was assessed using the Akaike information criterion (AIC, Akaike, 1974). Broad sense heritability estimates (H) were obtained on a genet mean basis by Bajgain et al. (2019a). Narrow‐sense heritability (*h^2^*) was estimated from the variance components obtained from each model in the following manner:

${h^{2}}_{A}={\sigma^{2}}_{A}/{\sigma^{2}}_{P}$ to obtain *h* ^2^ using additive effects
${h^{2}}_{\mathrm{AD}}=\left({\sigma^{2}}_{A}+{\sigma^{2}}_{D}\right)/\sigma^{2}$ to obtain *h* ^2^ using additive and dominance effects

where σ^2^_A_ is the additive genetic variance; σ^2^_D_ is the dominance genetic variance; and σ^2^_P_ is the phenotypic variance.

The additive relationship matrix was estimated using the *A.mat* function (Endelman, 2011). The dominance relationship matrix was estimated by assuming that the heterozygosity of an individual is contributing to dominance effects (Su, Christensen, Ostersen, Henryon, & Lund, 2012). The residual relationship matrix is assumed to contain epistatic effects, among others, and was estimated by considering only the second order epistasis and ignoring population inbreeding (Su et al., 2012). The latter assumption holds more true in the case of IWG because seed‐production through inbreeding in this plant is uncommon (Zhang et al., 2016). All estimates and calculations described in this section were carried out using the R package *sommer* (Covarrubias‐Pazaran, 2016).

### 2.3 Multi‐environment G×E models for genomic prediction

The UMN IWG breeding program evaluates its germplasm at two distinct MN locations for at least two years. Because of the inherent differences that exist between these sites as well as the expected genotype by year effect, models that could fit multi‐environment effects were tested in order to investigate their effect on trait predictions. Three multi‐environment genomic best linear unbiased prediction (GBLUP) models were tested, as described by Granato et al. (2018) in the R package *BGGE*:

1. MM, which considers only the main genetic effects in all environments
2. MDs, which considers the main genetic effect in all environments as well as a single G×E effect for each environment, and
3. MDe, which considers the main genetic effect in each environment as well as a single G×E effect for each environment.

The models MM and MDs incorporate genetic information from molecular markers and/or from environmental covariates (Jarquín et al., 2014) and model MDe decomposes marker in each specific environment as well as across all environments (Lopez‐Cruz et al., 2015). As these models have already been discussed in depth (Pérez‐Rodríguez et al., 2015; Bandeira e Sousa et al., 2017), they will not be described here further.

### 2.4 Evaluation of genomic prediction models using cross‐validation

Six different methods were used to predict the breeding values of the agronomic traits measured in UMN_C3 IWG breeding population: BayesA, BayesB, BayesC, Bayesian LASSO (BL), Bayesian Ridge Regression (BRR), and genomic best linear unbiased prediction (GBLUP). All Bayesian models were fitted as implemented in the R package *BGLR* (Pérez & de los Campos, 2014). GBLUP was implemented in the R packages *sommer* and *BGGE*.

In *sommer*, 1000 replications of the model were run with 20 iterations (maximum number allowed) in each run. For all models within *BGGE* and *BGLR*, 100 replications of each model were iterated 50,000 times with the first 25% discarded as burn‐ins and results sampled at every third iteration. Predictive ability of the models was obtained by calculating the correlation between observed phenotypic values and the genome estimated breeding values (GEBVs), that is, *r* = cor(PHENO_adj_, GEBV). Predictive abilities from all genomic prediction models were estimated using a four‐fold cross‐validation scheme. For this, UMN_C3 was randomly divided into four subsets and three out of the four subsets (338 genets) were used to train the models. The remaining subset with 113 genets was used as a validation set. This four‐fold cross‐validation scheme was replicated at least 100 times for all models in all packages. Table 1 summarizes all models and their components investigated by this study.

**TABLE 1:** Models and components used in genomic prediction of nine agronomic traits in UMN_C3 intermediate wheatgrass breeding population. Symbol ‘X’ means the component was used in the model. G×E, genotype by environment interaction effect

Method	Model	Effects		G×E	Tool	Replications	Iterations	Source
		Additive	Additive and Dominance					
GBLUP	General	X	X		*sommer*	1000	20	(Covarrubias‐Pazaran, 2016)
	MM	X	X	X				
	MDe	X	X	X	*BGGE*	100	50000	(Granato et al., 2018)
	MDs	X	X	X				
	BayesA	X	X					
	BayesB	X	X					
Bayesian	BayesC	X	X		*BGLR*	100	50000	(Pérez & de los Campos, 2014)
	BL	X	X					
	BRR	X	X					

### 2.5 Genetic gain from genomic selection

Expected genetic gain (ΔG) for trait ‘*y*’ was estimated using the following formula, as previously described by Heffner et al. (2010) and Rutkoski (2019):

$$\Delta G_{y}=ir\sigma_{A}$$

where *i* is the selection intensity, that is, trait selection differential (S) expressed in units of phenotypic standard deviation (σ_P_); *r* is the trait predictive ability obtained from genomic prediction models. For the sake of simplicity, only the highest *r* values observed among the three MM, MDe, and MDs models were used in the equation; and σ_A_ is the square root of additive genetic variance.

## 3 RESULTS

### 3.1 Variance components and trait heritability

**FIGURE 1:**
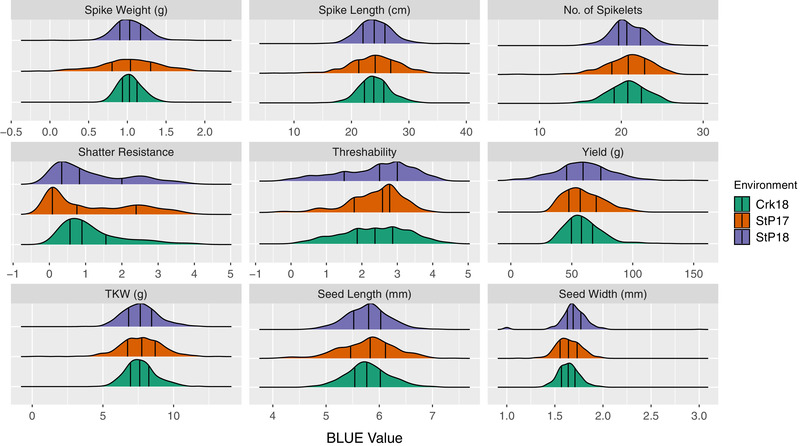
Density ridgeline plots of best linear unbiased estimates (BLUEs) for nine agronomic traits in the UMN_C3 intermediate wheatgrass breeding population. Traits were measured in three environments: Crookston, MN, USA in 2018 (Crk18), and Saint Paul, MN, USA in 2017 (StP17) and 2018 (StP18). Plot height within each trait facet is scaled for 0–1. Three vertical lines denote the four quantiles of data distribution

All traits discussed in this study exhibited quantitative distributions with occasional bi‐modality, such as in shatter resistance in Crk18 and StP17 environments and number of spikelets in StP18 environment (Figure 1). Variability among the environments was evident by different quantile data. Variance components and heritability estimates for all traits were obtained by fitting GBLUP mixed models on phenotype values adjusted for trial effects (PHENO_adj_) results are presented in Table 2. Calculations were carried out using components from models that accounted for additive effects only (A) and with both additive and dominance effects (AD). For all traits, the additive variance (σ^2^_A_) in the A model variant was equal to or larger than σ^2^_A_ estimates in AD models. Dominance variance estimates (σ^2^_D_) were larger than σ^2^_A_ in AD models for traits spike weight and seed width. Interestingly, σ^2^_D_ for number of spikelets, threshability, and grain yield were effectively zero. The largest proportion of the total genetic variance explained by σ^2^_A_ for any trait was for TKW in the A model (71%), and that by σ^2^_D_ for any trait was for seed width (52%). Overall, the AIC (Akaike information criterion) estimates showed that including dominance effects in the models improved model fitness relative to the A model, except for two traits: number of spikelets and threshability. For TKW, the A model was marginally better than the AD model. Yet, the differences observed in AIC estimates between A and AD model variants were small and not significant.

**TABLE 2:** Summary of additive (σ^2^_A_), dominance (σ^2^_D_), and residual (σ^2^_ε_) variance components along with narrow (*h^2^*) and broad‐sense (H) heritability estimates in the UMN_C3 intermediate wheatgrass breeding population. A, additive effects; AD, additive and dominance effects

Trait	Model	σ^2^_A_	σ^2^_D_	σ^2^_ε_	*h^2^*	H	AIC^a^
Spike weight (g)	A	0.010 (0.004)^b^		0.027 (0.003)	0.274 (0.088)		435.71
	AD	0.005 (0.009)	0.006 (0.009)	0.027 (0.003)	0.136 (0.233)	0.687 (0.094)	435.35
Spike length (cm)	A	2.971 (0.791)		4.104 (0.576)	0.420 (0.093)		419.89
	AD	1.559 (1.688)	1.516 (1.765)	3.946 (0.620)	0.222 (0.237)	0.438 (0.099)	419.17
Number of spikelets	A	1.368 (0.366)		1.902 (0.266)	0.418 (0.093)		393.97
	AD	1.368 (0.805)	0.000 (0.812)	1.902 (0.288)	0.418 (0.236)	0.418 (0.098)	393.97
Shatter resistance	A	0.388 (0.083)		0.292 (0.052)	0.570 (0.090)		333.87
	AD	0.227 (0.162)	0.187 (0.166)	0.264 (0.056)	0.334 (0.232)	0.610 (0.095)	332.24
Threshability	A	0.258 (0.065)		0.305 (0.045)	0.458 (0.093)		415.29
	AD	0.258 (0.139)	0.000 (0.139)	0.305 (0.049)	0.458 (0.235)	0.458 (0.098)	415.29
Yield (g)	A	41.915 (16.985)		145.070 (15.845)	0.224 (0.085)		440.18
	AD	41.921 (43.876)	0.000 (45.807)	145.065 (17.032)	0.224 (0.231)	0.624 (0.090)	440.18
Thousand kernel weight (g)	A	0.754 (0.135)		0.306 (0.073)	0.711 (0.081)		271.52
	AD	0.672 (0.251)	0.093 (0.243)	0.294 (0.080)	0.635 (0.217)	0.723 (0.086)	271.33
Seed length (mm)	A	0.086 (0.017)		0.053 (0.010)	0.618 (0.088)		335.83
	AD	0.062 (0.033)	0.029 (0.034)	0.049 (0.011)	0.442 (0.228)	0.649 (0.092)	335.02
Seed width (mm)	A	0.008 (0.002)		0.013 (0.002)	0.398 (0.093)		408.55
	AD	0.006 (0.005)	0.011 (0.005)	0.010 (0.002)	0.229 (0.162)	0.516 (0.098)	401.34

^a^AIC, Akaike information criterion;

^b^Values wihin parentheses are the standard error estimates for each component

The highest estimates of narrow sense heritability (*h^2^*) for all traits were observed in the A model variants (Table 2). The largest *h^2^* value was observed for TKW (0.71), followed by seed length (0.62) and shatter resistance (0.57). Broad sense heritability (H) estimates were larger than *h^2^* for all traits. The largest H values were observed for TKW (0.72) followed by spike weight (0.69) and seed length (0.65).

### 3.2 Trait predictions in additive and dominance models

**FIGURE 2:**
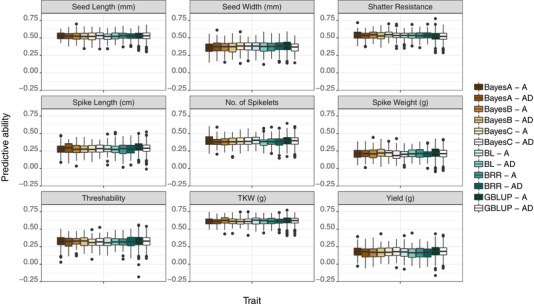
Distribution of trait predictive abilities estimated in the UMN_C3 intermediate wheatgrass population by Bayesian and GBLUP (genomic best linear unbiased prediction) models with additive only (“A”) and additive + dominance (“AD”) effects. BL, Bayesian LASSO; BRR, Bayesian Ridge Regression

**FIGURE 3:**
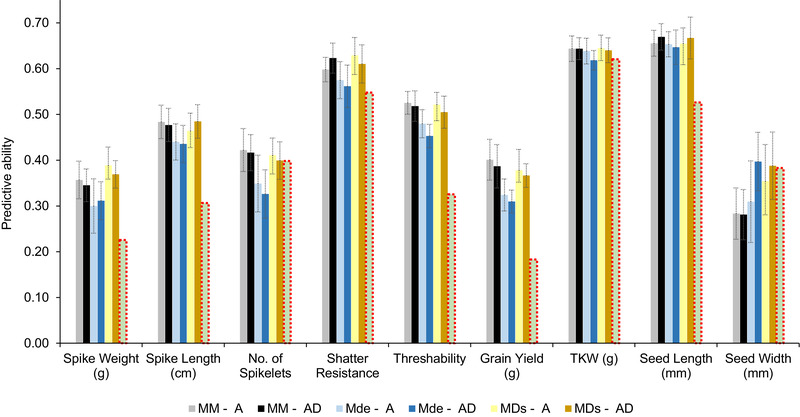
Predictive abilities (mean) observed when genotype by environment (G×E) interaction effects were fitted in genomic selection models MM, MDe, and MDs with their additive (“A”), and additive and dominance (“AD”) variants. Error bars represent the standard deviation of predictive ability values obtained from each model. Bars with dashed borders colored red represent the highest predictions for each trait obtained when G×E effects were not accounted for

As expected, different prediction methods produced dissimilar predictive abilities that varied by trait. While GBLUP models were generally better than Bayesian, no single method or model variant gave the highest predictive ability for any particular trait (Figures 2 and  3). One notable exception was the trait seed width where all Bayesian models performed relatively well, although not better than most other models. Models that incorporated G×E effects outperformed nearly all other models and model variants. The lowest predictive abilities were observed for multiple traits such as yield, spike weight, spike length, and threshability when G×E effects were not taken into consideration. Notable improvements in predictions with G×E included in the models were observed for yield, spike weight, spike length, and threshability with improvements of up to 19, 14, 19, and 19%, respectively. Between the A and AD variants of all models, neither was significantly better than the other for any given trait, except for seed width where a 9% increase in predictive ability was observed in the AD variant of MDs compared to its A variant.

### 3.3 G×E based genomic prediction

Trait predictions using the multi‐environment models MM, MDe, MDs showed that incorporating G×E effects can significantly boost predictive ability (Figure 3). Predictions for yield increased by more than two fold and predictions for spike weight, spike length, and threshability increased by at least 1.6 fold. The MDs model, which takes into account the main genetic effect in all environments and a single G×E effect for all environments, was overall better for most traits with the exception of spike length and grain yield for which MM had slightly better prediction results; TKW was predicted the same by both MM and MDs. For all traits, MDe under‐performed relative to MM or MDs. No significant differences in trait predictions were found between A and AD variations of MM, MDe, and MDs models. Although, the AD variant within MDe improved predictive ability of seed width from 0.31 to 0.40, an increase by 9%. In MM, the AD version of the model was better for shatter resistance and seed length; for MDe, the AD model was better for two traits: spike weight and seed width; and for MDs, the AD model was better for three traits: spike length, seed length, and seed width.

### 3.4 Cross‐environment predictions

**FIGURE 4:**
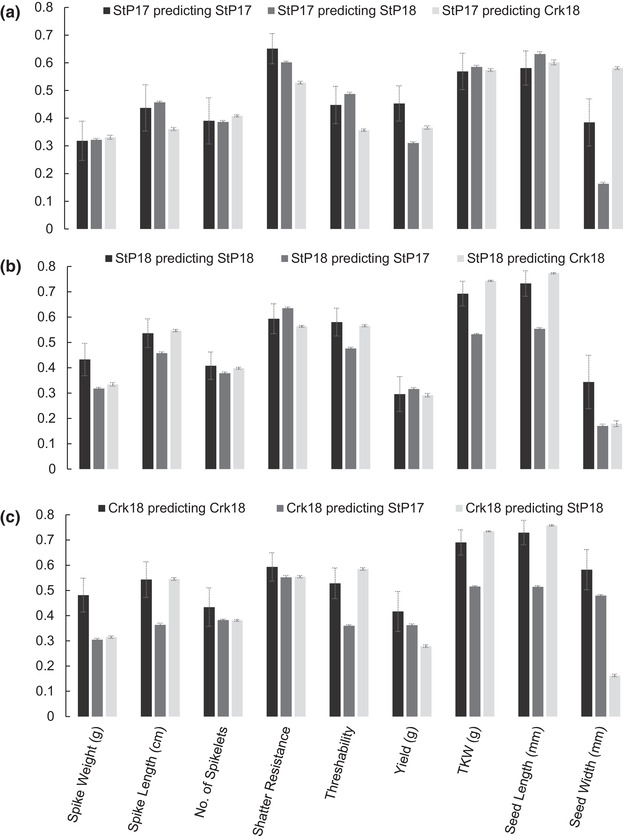
Cross‐environment genomic predictions from traits measured in three environments: Crookston, MN, USA in 2018 (Crk18), and Saint Paul, MN, USA in 2017 (StP17) and 2018 (StP18). Predictions were using (a) StP17 to predict StP18 and Crk18, (b) StP18 to predict StP17 and Crk18, and (c) Crk18 to predict StP17 and StP18. Predictive abilities were obtained from 100 replicates (50000 iteration of each replicate) of the additive variant of the MDs model (“MDs – A”). Traits are displayed on the x‐axis and predictive abilities are displayed on the y‐axis

The additive variant of the MDs model (MDs‐A) was trained on single environment data from which GEBVs were predicted for the remaining two environments, i.e. using StP17 to predict StP18 and Crk18, StP18 to predict StP17 and Crk18, and Crk18 to predict StP17 and StP18. A four‐fold validation approach was used here as well where a random 75% subset of a population in an environment was used to predict remaining 25% within the environment, and the same 75% was used to predict the other two environments. This process was replicated 100 times. MDs‐A model was the overall best performer, and results are summarized in Figure 4. The highest cross‐environment predictions were observed for seed length when StP18 data were used to predict Crk18 (*r* = 0.77) and Crk18 data were used to predict StP18 (*r* = 0.76). Overall, the prediction estimates ranged from 0.16 to 0.77 and no one environment was the overall best predictor of all traits in the remaining two environments. The first year trial (StP17) was the best predictor of both second year trials for spike weight, number of spikelets, TKW, and seed length with predictive abilities that deviated ≤ 4% from the mean predictions. StP18 was the best predictor for grain yield in all environments with predictions of 0.30, 0.32, and 0.29 in StP18, StP17, and Crk18, respectively. Crk18 was not a good predictor of either Saint Paul environments.

### 3.5 Genetic gain from prediction models

Expected genetic gain estimated by the A models for all traits ranged 0.02‐1.88 and 0–1.82 for the AD models (Table 3). The highest gain predicted based on genomic prediction models was observed for grain yield where both A and AD models estimated an improvement of nearly two units relative to genetic standard deviation. For all traits except number of spikelets and threshability, the A models showed higher gain than the AD model. Seed width had the smallest gain estimates (< 0.02) in both A and AD models followed by spike weight and seed length.

**TABLE 3:** Expected genetic gain (ΔG = *ir*σ_A_) in the UMN_C3 intermediate wheatgrass breeding population based on multi‐environment genomic prediction models. ΔG values are shown in percentage per breeding cycle (2 years)

Trait^a^	σ^2^_A_‐A	σ^2^_A_‐AD	σ_P_	i	*r*_A_	*r*_AD_	ΔG_A_	ΔG_AD_
Spike weight (g)	0.01	0.01	0.26	1.03	0.39	0.37	0.03	0.02
Spike length (cm)	2.97	1.56	3.30	0.83	0.48	0.48	0.20	0.14
No. of spikelets	1.37	1.37	2.44	1.04	0.42	0.42	0.07	0.07
Shatter resistance	0.39	0.23	1.03	1.07	0.63	0.62	0.13	0.10
Threshability	0.26	0.26	0.91	1.17	0.53	0.52	0.13	0.13
Grain yield (g)	41.92	41.92	18.06	0.95	0.40	0.39	1.88	1.82
Thousand kernel weight (g)	0.75	0.67	1.31	1.04	0.64	0.64	0.76	0.72
Seed length (mm)	0.09	0.06	0.45	1.01	0.66	0.67	0.12	0.11
Seed width (mm)	0.01	0.00	0.14	1.10	0.35	0.40	0.02	0.00

^a^σ^2^_A_‐A and σ^2^_A_‐AD, additive variance calculated using additive only and additive + dominance models, respectively; i, selection intensity; σ_P_, phenotypic distribution standard deviation; *r*_A_ and *r*_AD_, highest predictive abilities obtained in models with additive only and additive + dominance effects, respectively; ΔG_A_ and ΔG_AD_, expected genetic gain in models with additive only and additive + dominance effects, respectively.

## 4 DISCUSSION

Genomic selection (GS) is an alternative selection approach that can help breeders make sound breeding decisions and increase genetic gain per unit time and cost (Meuwissen et al., 2001; Heffner, Sorrells, & Jannink, 2009). In silico selections carried based on GS models cost a fraction relative to multi‐location and multi‐year phenotypic evaluations. As the cost of high‐throughput genotyping continues to decline, several thousand high quality genome‐wide polymorphic markers, primarily SNPs, can be discovered in any plant or animal species and make GS‐based breeding an attractive option. In fact, the implementation of GS in our IWG breeding program has trimmed 3–4 years from our conventional variety development pipeline. One of the basic objectives of our GS based IWG breeding approach is to carry out timely training, updating, and fine‐tuning of existent GS models as new and promising algorithms become available. In this study, we provide the results obtained from training several GS models that include additive and non‐additive effects and G×E interaction effects with cross‐site predictions.

In our phenotypic data, we observed that additive effects have a bigger role in dictating the performance of most IWG traits, yet the contribution from non‐additive effects cannot be completely ignored. We saw substantial contributions from dominance and higher order genetic effects, possibly epistatic, in the phenotypic variance of a few traits (Table 2). For instance, the dominance effect contributed an additional 1.2‐ to 2‐fold the additive variance for spike weight, spike length, and shatter resistance in AD models. While these contributions are mostly of small magnitude, AIC estimates suggested that including dominance effects can improve overall model fitness. Our results also suggest that the distribution of additive and non‐additive effects in IWG are highly trait specific and are possibly affected by the diverse environments where our trials are conducted. Another interesting observation is that the dominance variance was often either zero or of negligible value in AD model variants for number of spikelets, yield, threshability, seed length, and TKW. These traits therefore likely do not rely on dominance for trait expression. We noticed that heritability estimates for several traits were not constant among these three model variants. Narrow sense heritability (*h^2^*) estimates for most traits reduced by 11–70% when dominance and higher order genetic effects were partitioned. There were some exceptions: no change in heritability was seen for number of spikelets, threshability, and grain yield between the A and AD model variants. Lower estimates of *h^2^* are not uncommon in non‐additive models and has been shown to be the case in both plant and animal species (Su et al., 2012; Bouvet, Makouanzi, Cros, & Vigneron, 2016). This is because a large portion of non‐additive genetic variance is often included within the additive variance in A model when no further partitioning of variance components is done, giving higher estimates of *h^2^*. Broad sense heritability (H) is often larger than *h^2^* because all genetic variance is included in the numerator (Falconer & Mackay, 1996).

We observed that GBLUP models were generally better than Bayesian models whether or not G×E effects were fitted in the models. Seed width was the only trait that deviated from this pattern as nearly all Bayesian models had predictions similar to the GBLUP models. For spike morphology such as spike weight, spike length, number of spikelets, as well as grain yield, a large amount of genetic variance was neither additive nor dominance. Depending on the trait and the model variant, the residual variances for these traits were nearly 1.4‐ to 5.4‐fold larger than σ^2^_A_ or σ^2^_D_. Thus, it became apparent that modeling only the additive and dominance variance is often inadequate in accurate prediction of GEBVs for several traits. Therefore, we decided that G×E must be taken into account in order to obtain predictions that are more reliable. On the other hand, in the discussed IWG traits, non‐additive effects appear to have not‐so‐significant contributions to genetic variance components as well as in genomic prediction. This was the case even for traits such as spike weight, spike length, and shatter resistance where the dominance effect was 1.2‐ to 2‐fold larger relative to additive variance. Predictions improved by up to 3% but overall were small increases. This increase is similar to that observed in other crop species where fitting dominance effects in the prediction models only modestly increased the predictions (Wolfe, Kulakow, Rabbi, & Jannink, 2016; Morais et al., 2017). Hence, while dominance effects could be modeled in IWG trait prediction to improve predictions by a small factor, simply using additive models with G×E effects will provide similar results.

Indeed, fitting G×E interaction effects in GS prediction models has shown to be more effective than univariate prediction models and improved trait predictions in several crop species (Ly et al., 2013; Lopez‐Cruz et al., 2015; Wang et al., 2016). The IWG breeding population presented in this study was phenotyped at two diverse MN locations. Because of location and year effects as observed from the variance component calculations, modeling G×E interaction effects in GS models provided a significant boost in trait predictions. In particular, the models MDe and MDs were vastly superior to any other models we tested, for all traits. These two models include a kernel for main genetic effect as well as additional kernels for G×E effects within and/or across the different environments. This is likely due to the prediction models benefiting by extracting information on trait performance of the same genotypes from multiple environments (Granato et al., 2018). We then used these models to predict trait values in two environments using data collected in one environment. While predictions varied by trait, those with higher heritabilities and low residual environmental variance were often predicted better by using one environment data (Figure 3). This is expected as traits are influenced by different environmental variables in different locations and years. The results indicate that traits with high heritabilities can be effectively selected using the first year in Saint Paul environment (StP17). This is an important finding for us as phenotyping IWG in multiple locations is resource and labor‐intensive. Therefore, being able to predict the performance of some agronomic traits in independent locations using data collected in one environment would speed our IWG domestication efforts.

An increase in trait predictive ability can improve selection accuracy and overall breeding efficiency, especially for complex traits such as yield and seed characteristics that are typically affected by a large number of genetic and environment factors (Jarquín et al., 2014). This was corroborated in our analysis as we obtained relatively high genetic gain estimates in our trials. The largest gains were estimated for grain yield (1.88 units or 34.0 g increase per breeding cycle on average) and TKW (0.76 or 1.0 g increase per breeding cycle), which is expected since both these traits are under strong selection pressure in our breeding program. UMN_C4 is currently being phenotyped and we will carry out a follow up study to see how these estimated gain predictions compare with realized gains based on field phenotyping. We are equally interested in tracking the selection responses from one generation to the next as well as the amount of genetic variance in our current and future breeding germplasm. In self‐pollinated crops, evidence suggests that GS‐based breeding can cause a significant decline in genetic variance compared to conventional breeding approaches (Gaynor et al., 2017; Muleta, Pressoir, & Morris, 2019). The case of IWG is different in that it is an out‐crossing species with three large sub‐genomes with some level of gene redundancy. Does being an out‐crossing hexaploid have any advantage in slowing down the reduction of genetic variance observed in inbred cereal crops? We plan to investigate this by evaluating the parents of all breeding cycles in a ‘common garden’ experiment.

To summarize, as a perennial grain species with a long cropping cycle, IWG can greatly benefit from GS prediction models that consider G×E effects. This can potentially help in developing elite varieties with stable and better performance in multiple environments. Our findings discussed herein are encouraging in rapid domestication and improvement of IWG as these approaches promise higher genetic gains from modeling of G×E interaction effects. The methods and results presented in this study will likely be useful to not only IWG breeding, but also other crops that are undergoing domestication and need rapid trait improvement.

## CONFLICT OF INTEREST

The authors declare no conflict of interest.
